# Supplementary material for: Reconfiguration of Gut Microbiota and Reprogramming of Liver Metabolism with Phycobiliproteins Bioactive Peptides to Rehabilitate Obese Rats
Source: Nutrients. 2022 Sep 2;14(17):3635. doi: 10.3390/nu14173635 (PMC9460120; doi:10.3390/nu14173635)
Supplement: Supplementary file 1 [file nutrients-14-03635-s001.zip › nutrients-1852248-supplementary.pdf]

Table S1. The primer sequences used in this study.

Gene	Sequence (5’ to 3’)	
β-actin	Forward	CCCGCGAGTACAACCTTCTTG
	Reverse	ACCCATACCCACCATCACAC
GAPDH	Forward	GCAAGTTCAACGGCACAG
	Reverse	GCCAGTAGACTCCACGACAT
IRS1	Forward	ATGTCGCCAGTGGGAGATT
	Reverse	CTTCGGCAGTTGCGGTATA
IRS2	Forward	CAGCACCTACGCAAGCATCG
	Reverse	CCGCAGCACTTTACTCTTTCACC
Akt2	Forward	TTCTACAACCAGGACCACGAGC
	Reverse	TGATGCTGAGGAAGAACCGATG
Pi3K	Forward	CCTCTCCTTATAAAGCTCCTGGAA
	Reverse	GATCACAATCAAGAAGCTGTCGTAA
Gsk3β	Forward	TTAAGGAAGGAAAAGGTGAATCGA
	Reverse	CCAAAAGCTGAAGGCTGCTG
Gys	Forward	TCCTCAGTACCACCTTCC
	Reverse	AGCCTCCTCTTCCTCATC
FBP	Forward	GTCCTATGCTACCTGTGTC
	Reverse	TTCTCCGAAGGCTCATTAG
G6Pase	Forward	GAAGGCCAAGAGATGGTGTGA
	Reverse	TGCAGCTCTTGCGGTACATG
PEPCK	Forward	CCCAGGAAGTGAGGAAGTTTGT
	Reverse	GGAGCCGTCGCAGATGTG
FoxO1	Forward	GCATCCATGGACAACAACAG
	Reverse	TGATGGTGCTAGCGTTTGAG
CD36	Forward	AGGCTGTTGAGCACACCTTGAAC
	Reverse	TGAGCCTTCACTGTCTGTTGGAAC

ACC	Forward	CAATCCTCGGCACATGGAGA
	Reverse	GCTCAGCCAAGCGGATGTAGA
FASN	Forward	CAGGTGTGTGATGGGAAG
	Reverse	TGTGGATGATGTTGATGATAG
CPT1	Forward	CCCTAAGCCCACAAGGCTAC
	Reverse	TCTCTGTCCTCCCTTCTCGG

Table S2. The weight changes of rats during establishment of obesity model.

Group	Initial weight (g)	Obesity model weight (g)	Number of rats
Chow diet control group	201.33 ± 3.14	447.83 ± 19.91	6
High fat diet model group	202.48 ± 4.01	538.81 ± 24.86 **	24

Compared with chow diet control group ** p<0.01

Table S3. The serum triglyceride (TG) and total cholesterol (TC) contents (mmol/L) of rats during establishment of obesity model.

Group	TG contents (mmol/L)	TC contents (mmol/L)	Number of rats
Chow diet control group	0.808 ± 0.017	2.269 ± 0.028	6
High fat diet model group	0.913 ± 0.022*	2.413 ± 0.025	24

Compared with chow diet control group * p<0.05

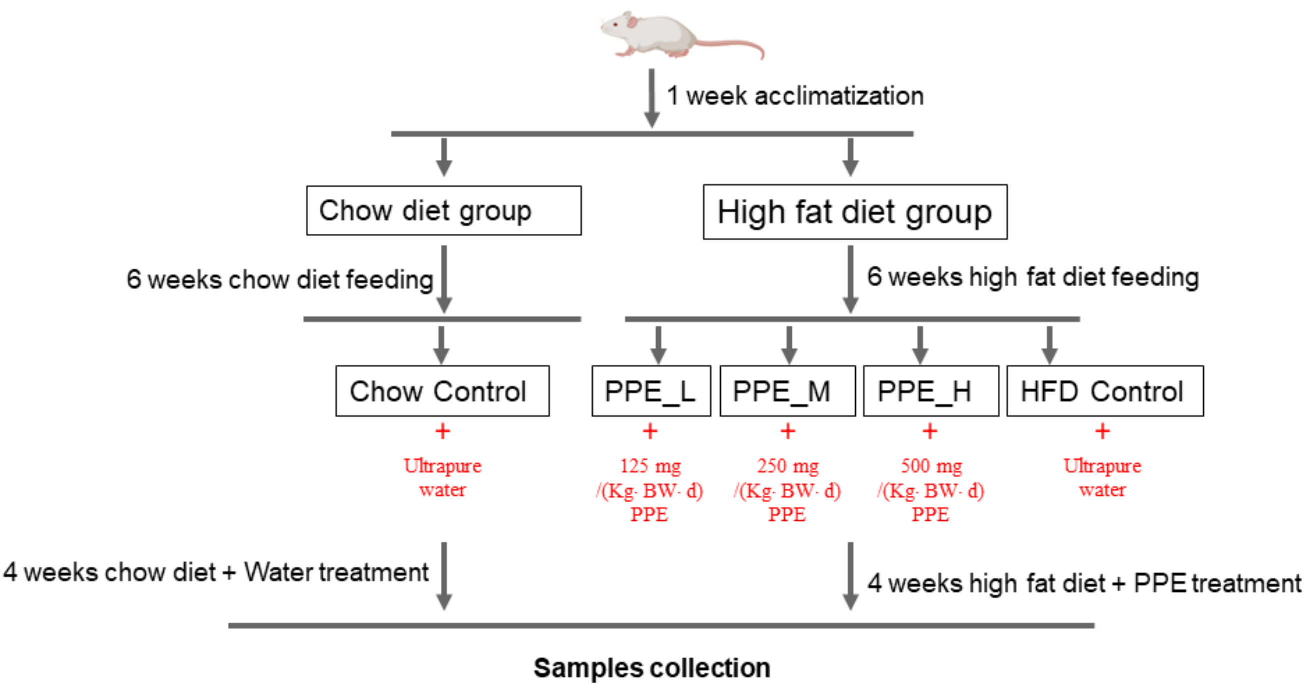

Figure S1. The experimental schedule and grouping information.

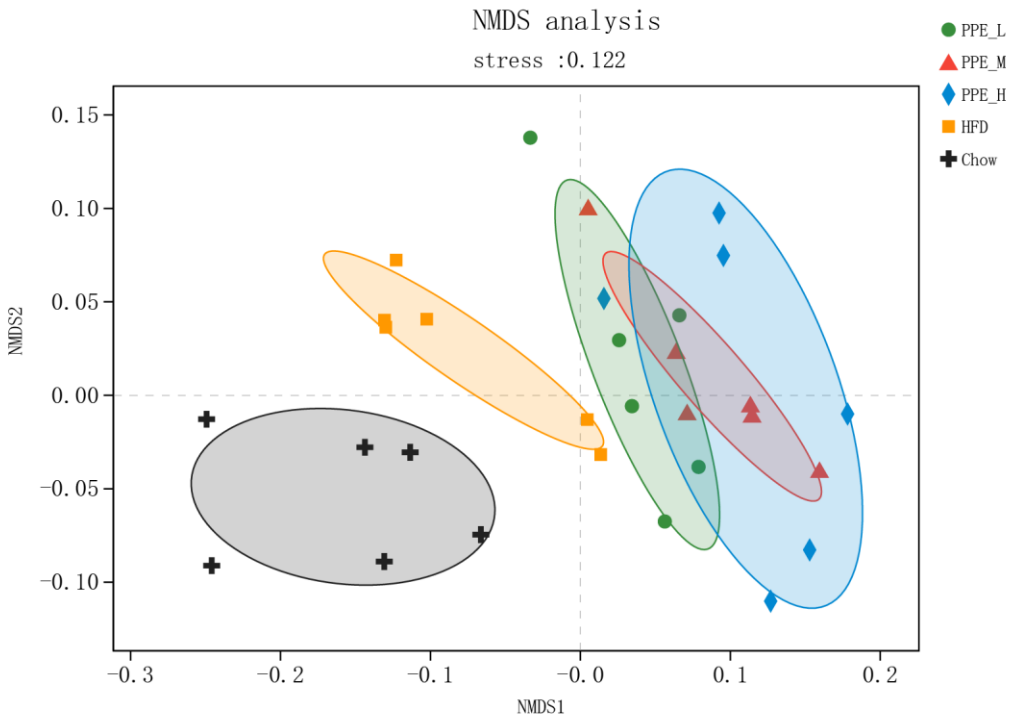

Figure S2. Gut microbiota composition in rats from different groups assessed by NMDS.

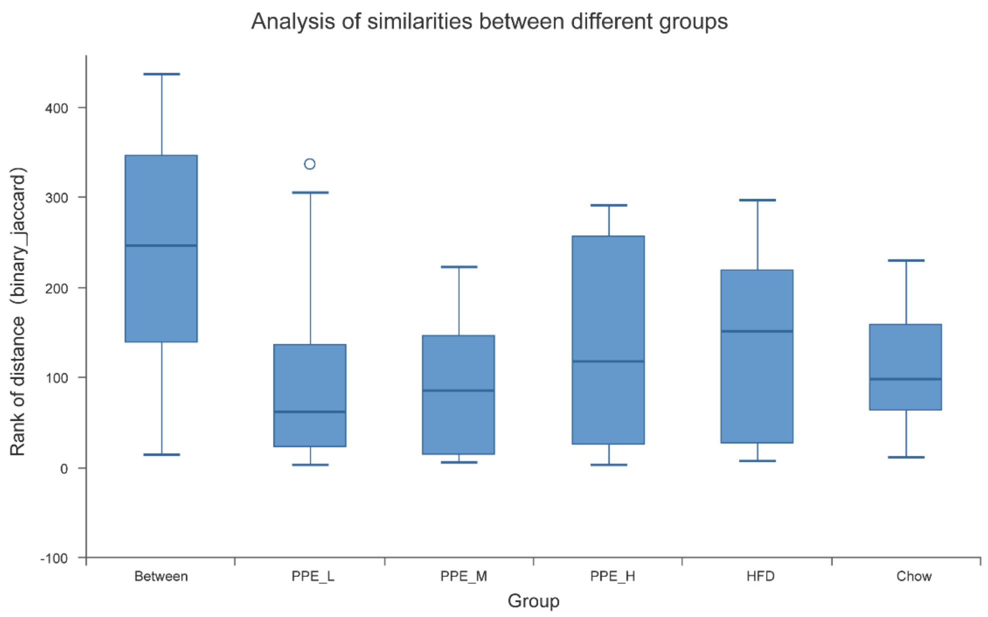

Figure S3. Gut microbiota composition in rats from different groups assessed by ANOSM.

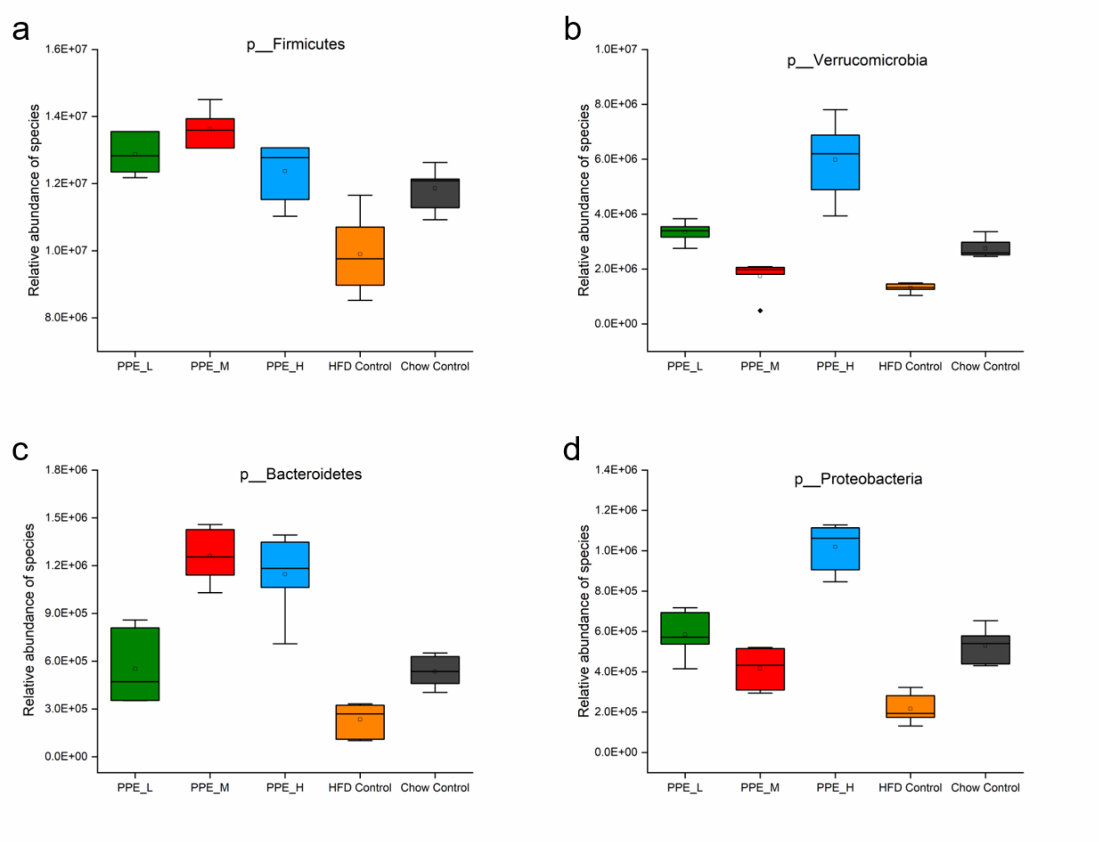

Figure S4. PPE modulate gut microbiota composition at the phylum level. Firmicutes (a),

Verrucomicrobia (b), Bacteroidetes (c) and Proteobacteria (d).

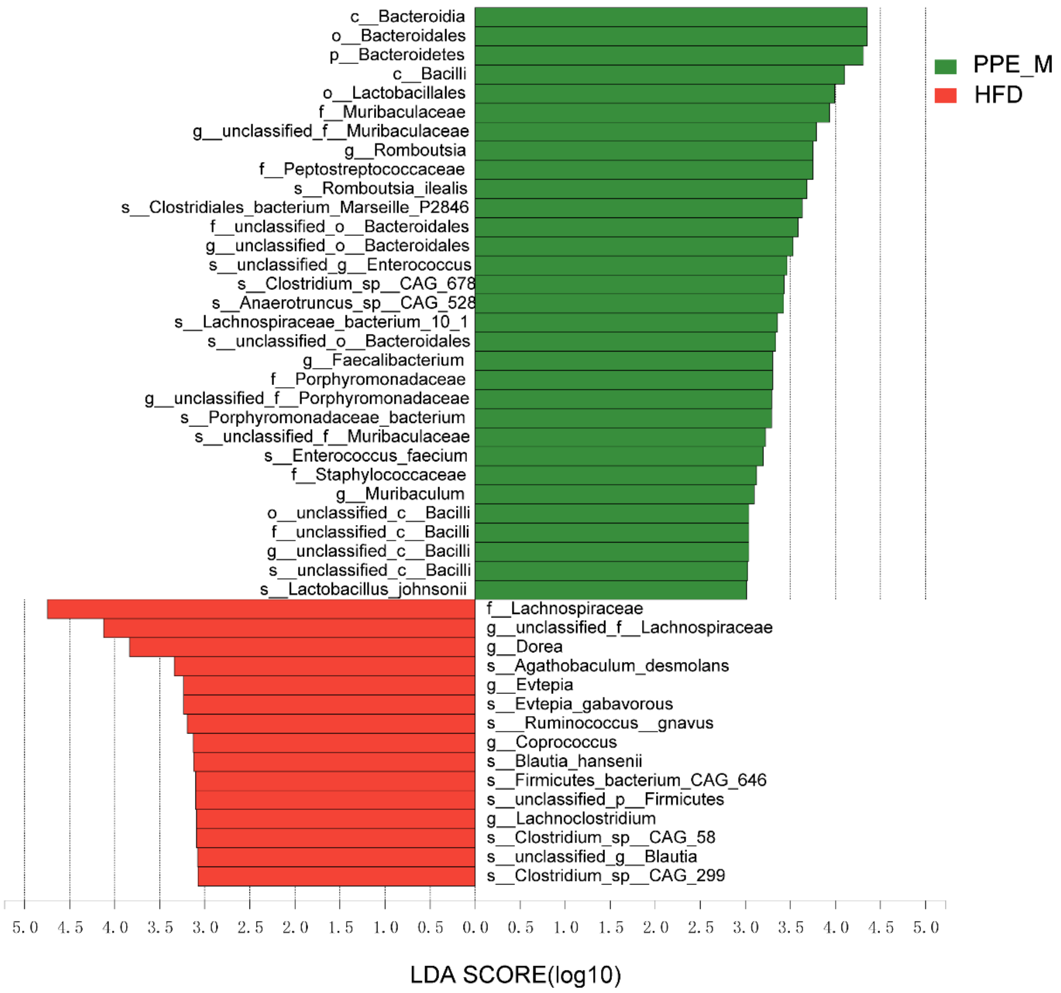

Figure S5. Cladogram obtained from LEfSe analysis (LDA > 3), showing the most differentially abundant taxa enriched in microbiota from rats in PPE_M (green) and HFD control (red) groups.

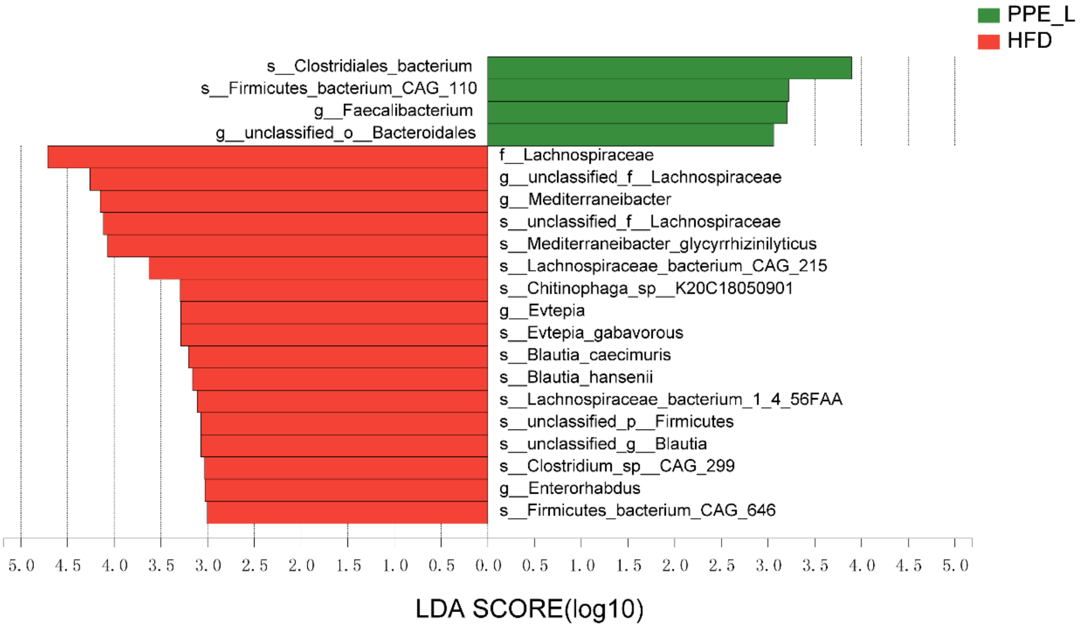

Figure S6. Cladogram obtained from LEfSe analysis (LDA > 3), showing the most differentially abundant taxa enriched in microbiota from rats in PPE_L (green) and HFD control (red) groups.

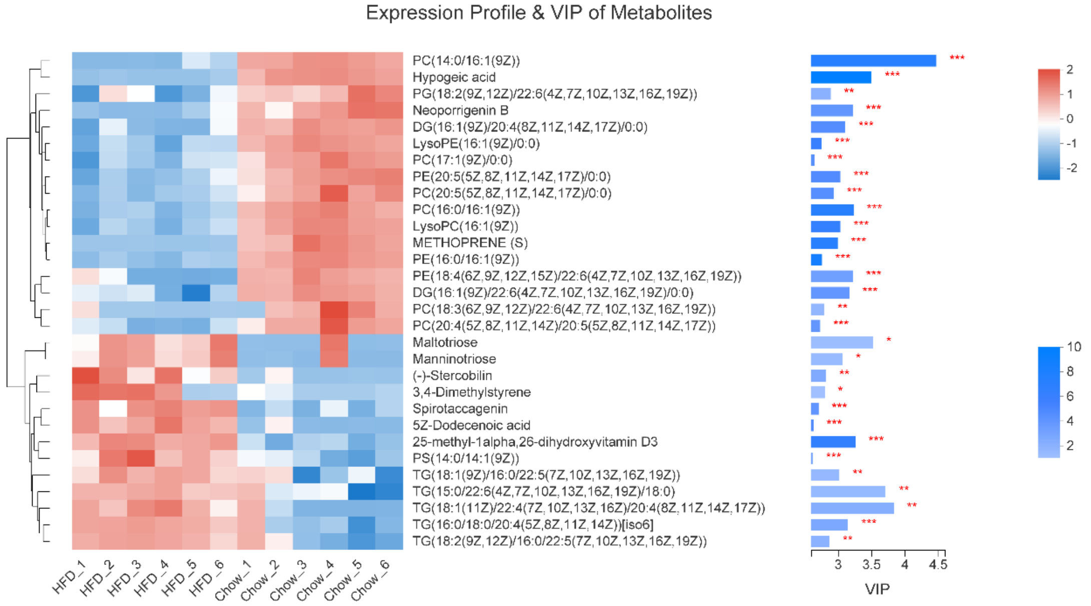

Figure S7. Expression profile and VIP score (OPLS-DA) of significantly different metabolites (top 30 are shown, * represents P<0.05, ** represents P<0.01, * represents P<0.001) between Chow diet & HFD diet (n = 6 rats in each group).**

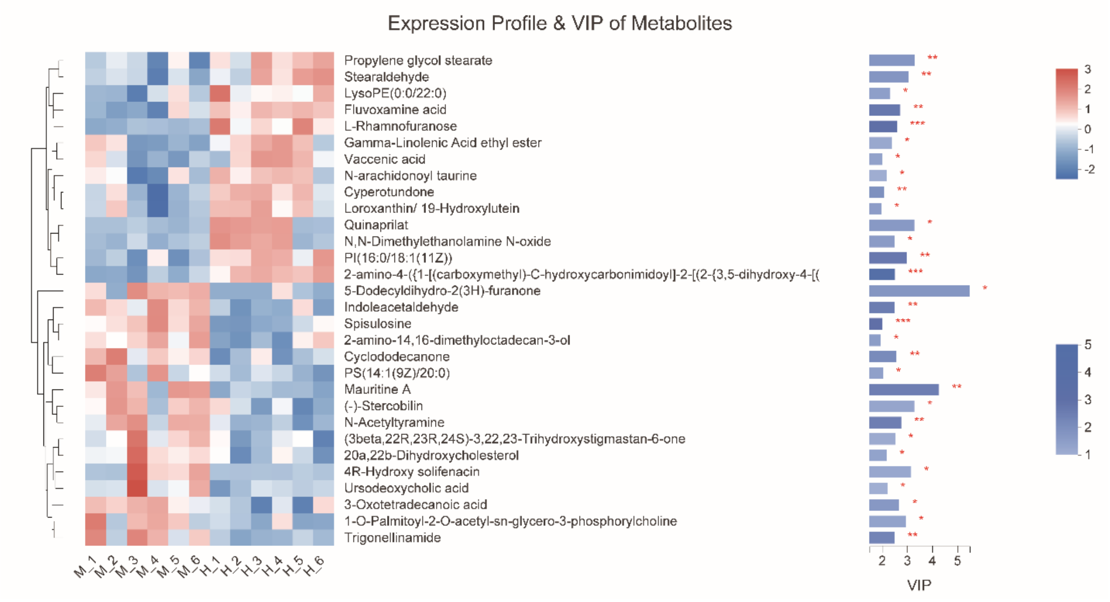

Figure S8. Expression profile and VIP score (OPLS-DA) of significantly different metabolites (top 30 are shown, * represents P<0.05, ** represents P<0.01, * represents P<0.001) between PPE_M diet & HFD diet (n = 6 rats in each group).**

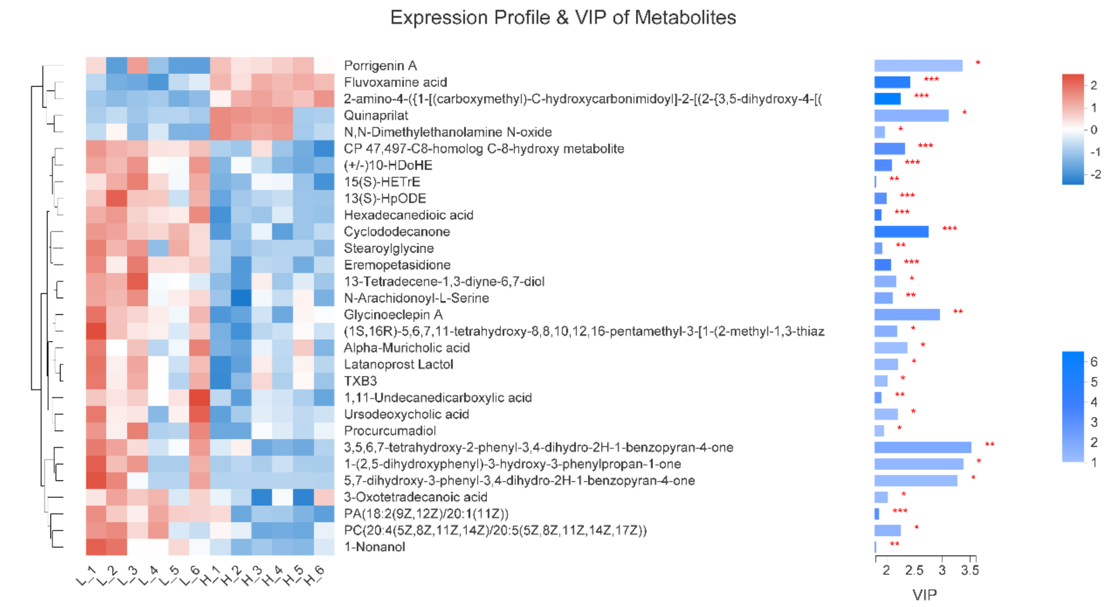

Figure S9. Expression profile and VIP score (OPLS-DA) of significantly different metabolites (top 30 are shown, * represents P<0.05, ** represents P<0.01, * represents P<0.001) between PPE_M diet & HFD diet (n = 6 rats in each group).**

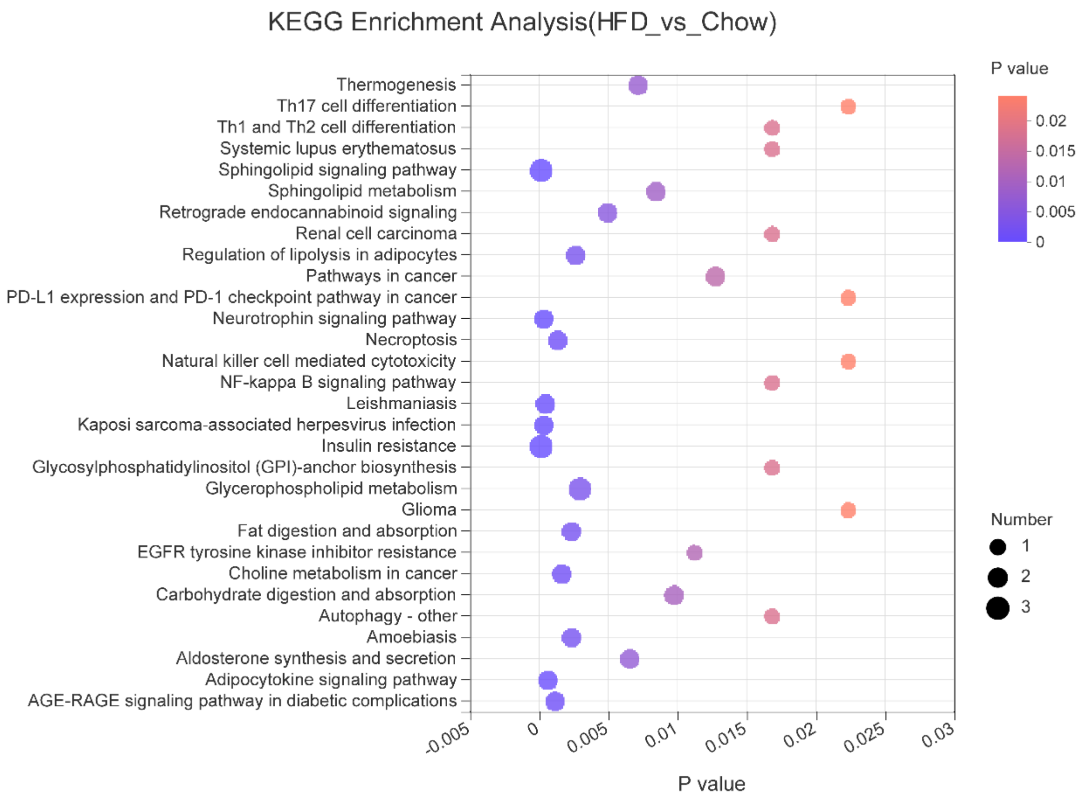

Figure S10. KEGG enrichment analysis (top 30 are shown) between PPE_M diet & HFD diet between Chow diet & HFD diet. (n = 6 rats in each group).

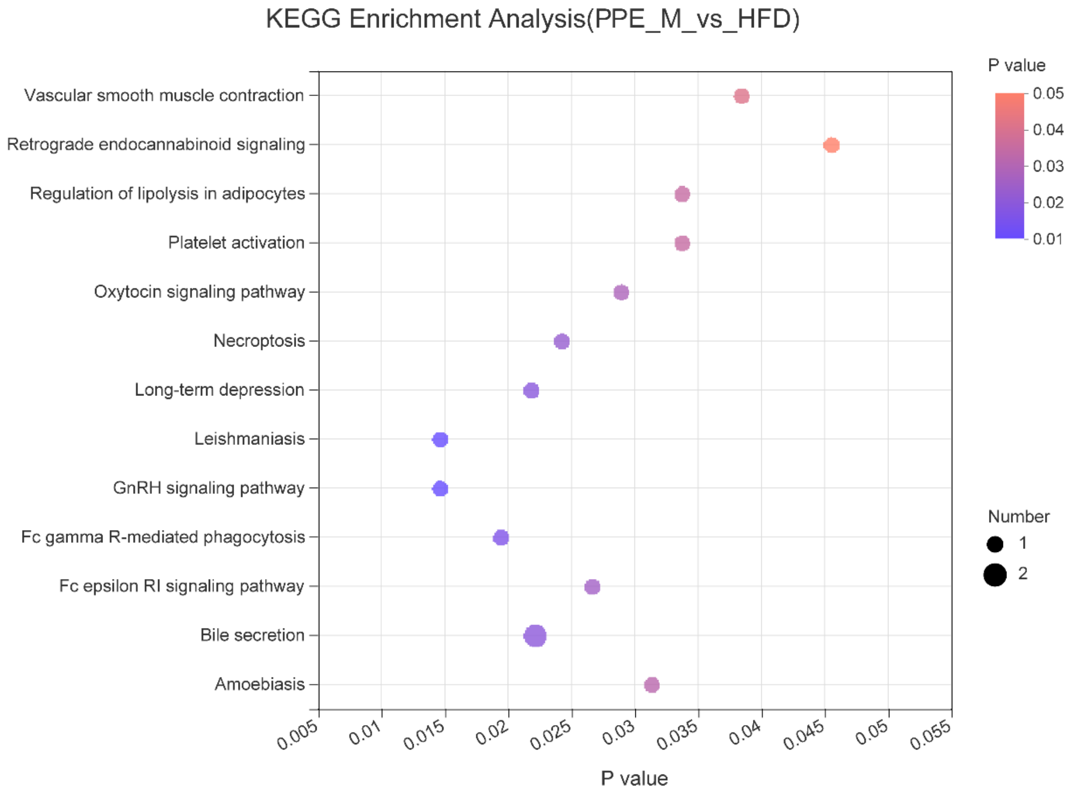

Figure S11. KEGG enrichment analysis (top 30 are shown) between PPE_M diet & HFD diet between PPE_M diet & HFD diet. (n = 6 rats in each group).

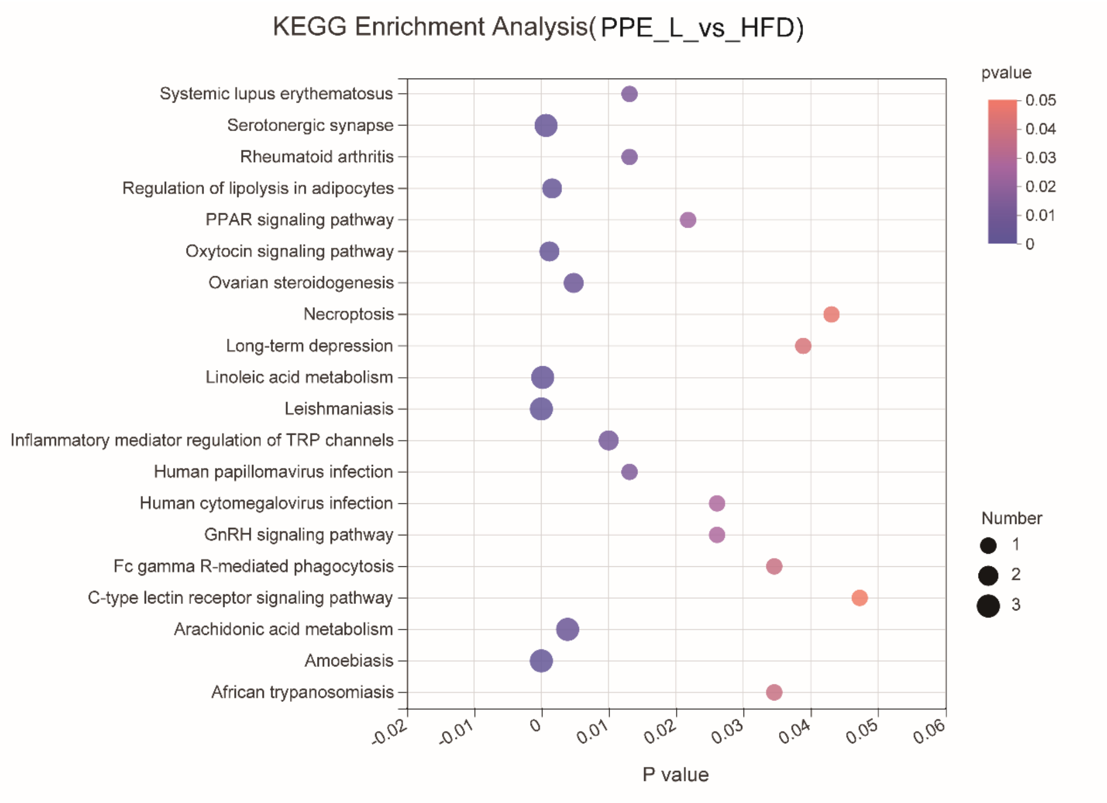

Figure S12. KEGG enrichment analysis (top 30 are shown) between PPE_M diet & HFD diet between PPE_L diet & HFD diet. (n = 6 rats in each group).
